# Leaf Morpho-Anatomy of Twelve *Cymbidium* (Orchidaceae) Species from China and Their Taxonomic Significance

**DOI:** 10.3390/plants14091396

**Published:** 2025-05-06

**Authors:** Xiangke Hu, Lei Tao, Jialin Huang, Kaifeng Tao, Dong Ma, Lu Li

**Affiliations:** 1 College of Forestry, Southwest Forestry University, Kunming 650224, China; hxk1999@swfu.edu.cn (X.H.); tl0427@126.com (L.T.); 17856109959@163.com (K.T.); md2549256739@163.com (D.M.); 2 School of Chemistry, Biology and Environment, Yuxi Normal University, Yuxi 653100, China; hjl@yxnu.edu.cn; 3 Yunnan Academy of Biodiversity, Southwest Forestry University, Kunming 650224, China

**Keywords:** *Cymbidium*, cuticle, fiber bundle, leaf epidermis, stegmata, stigmata, stomata, outline of blade cross-section

## Abstract

*Cymbidium* are endangered and ornamental orchids, and the taxonomy and species identification of this genus have been debated due to some overlapping morphological features between taxa and limited data being available. The leaf morpho-anatomy of 12 *Cymbidium* species from China was investigated using light microscopy and paraffin sectioning. Based on a comparative analysis, some leaf morphological features that varied between species were selected and used for taxonomic differentiation as follows: (1) The shape and structure of leaves were varied and could be used for species delimitation. (2) Microscopic characteristics show that the leaves lacked trichomes and displayed polygonal to rectangular epidermal cells on both surfaces, with larger adaxial cells and more abaxial stigmata. Stomata were mostly distributed only on the abaxial side, but on both sides in *Cymbidium mastersii*, which exhibited a rare amphistomatic type. The stomatal complex was uniformly tetracytic in 11 species, while it was observed to be anomocytic in *C. floribundum*. (3) Anatomically, two distinct midrib configurations were identified, a shallow V-shape and V-shape. The mesophyll cells were homogeneous in 10 species, with the exception of a layer of parenchyma cells resembling palisade cells occurring in *C. lancifolium* and *C. qiubeiense*. The thickness of the cuticle varied between species, with the adaxial surface covered by a thicker cuticle than the abaxial surface and displaying either a smooth or corrugated surface. A fiber bundle was observed in six species, but absent in the other six. In the former group, the fiber bundle occurred adjacent to both epidermal cells in *C. mastersii* and *C. hookerianum*, while it was adjacent to the abaxial epidermis in four other species. The stegmata, with conical, spherical silica bodies, were associated with fiber bundles and mesophyll in seven species, but absent in the other five (*C. kanran*, *C. defoliatum*, *C. floribundum*, *C. lancifolium*, and *C. serratum*). Three kinds of crystals were identified, namely the terete bundle, the long tube bundle, and the raphide. (4) It was suggested that some of these variable features could be selected and used for the delimitation of the species and taxonomy of *Cymbidium*. In addition, a key to the 12 *Cymbidium* species based on their leaf morpho-anatomic features was proposed, which could lead to a better understanding of the taxonomy and conservation of Orchidaceae.

## 1. Introduction

*Cymbidium* Sw. (Orchidaceae), comprising about 52 species, is predominantly found in the tropical and subtropical regions of Asia, with some species extending to Papua New Guinea and Australia [1]. There are around 49 species (19 endemic) that grow in China, the distribution center of *Cymbidium* [2]. *Cymbidium* species demonstrate diverse ecological adaptations such as epiphytic, lithophytic, or terrestrial orchids, which are characterized by ovoid to spindle-shaped pseudobulbs, distichous narrowly ligulate to elliptic leaves, showy and often fragrant flowers with trilobed labella, and two or four pollinia [1,3]. The species of *Cymbidium* found in China, known as ‘Guolan’ in Chinese, have been widely cultivated for over two hundred years due to their high ornamental value [2,3]. However, wild populations of *Cymbidium* have experienced a significant decline due to overexploitation and habitat fragmentation and are listed as rare and endangered species [4,5,6,7].

The infrageneric taxonomy of *Cymbidium* (the category of a section or subgenus of the genus and identification of different species) has been problematic and controversial due to the addition of newly described species [1,2,8,9,10]. Three subgenera of *Cymbidium* have been identified—subgen. *Cymbidium* (L.) Sw., subgen. *Cyperorchis* (Bl.), and subgen. *Jensoa* (Rafin.)—based on their pollinium number and labellum–column fusion [11]. This classification system has been adopted by several subsequent taxonomists [1,2,9]. Previously, 29 were species recorded in China, which were classified into three subgenera containing 14 sections [3]. Later, the number of *Cymbidium* species identified increased to 68 species, and the genus was classified into three subgenera containing with 16 sections, with 49 of these species found in China [9]. Later, *Cymbidium* was thought to contain 52 species and was divided into 11 sections based on molecular data from ITS and *matK* [1]. In addition, another study recognized 55 *Cymbidium* species (including the 49 from China), without further classifying them into subgenera or sections [2].

**Figure 1:**
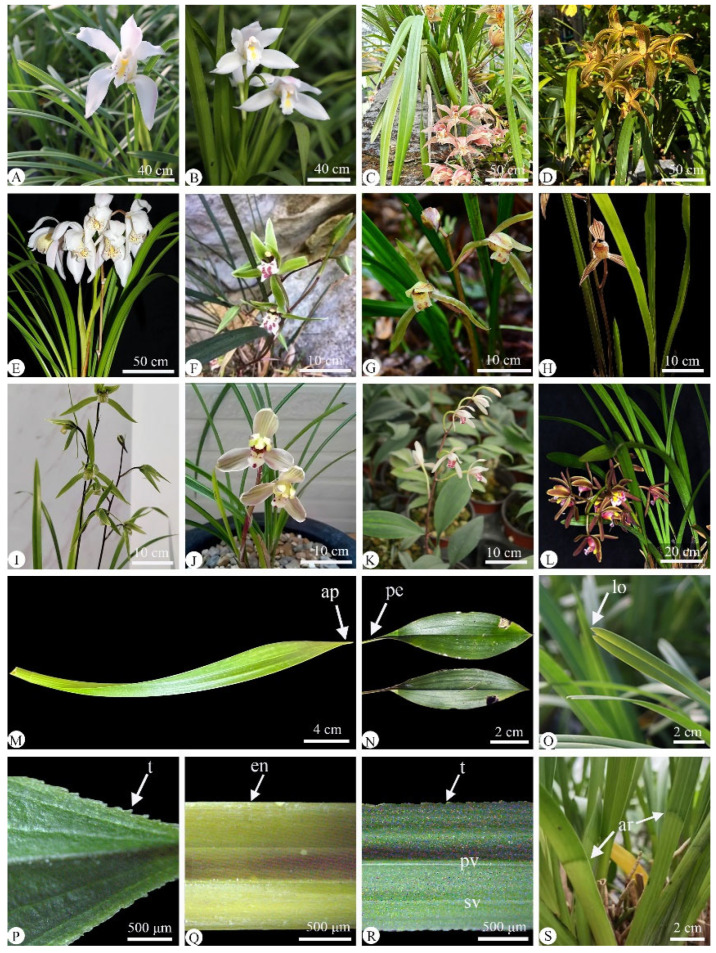
Plants of twelve *Cymbidium* species, showing their flower, leaf morphology and detailed leaf features. (**A**–**L**) Flower and leaf of twelve *Cymbidium* species; (**M**–**S**) detailed leaf features; (**A**–**E**) subgen. *Cyperorchis*; (**F**–**K**) subgen. *Jensoa*; (**L**) subgen. *Cymbidium*. (**A**) *C. eburneum*; (**B**) *C. mastersii*; (**C**) *C. hookerianum*; (**D**) *C. tracyanum*; (**E**) *C. wenshanense*; (**F**) *C. qiubeiense*; (**G**) *C. faberi*; (**H**) *C. defoliatum*; (**I**) *C. kanran*; (**J**) *C. serratum*; (**K**) *C. lancifolium*; (**L**) *C. floribundum*; (**M**) Lorate leaf of (**L**) *C. floribundum* with slightly oblique apex (enlarged image); (**N**) oblanceolate–oblong leaf; (**O**) leaf apex and two-lobed leaf; (**P**) leaf of (**N**) *C. lancifolium* (enlarged image) with apex margin and finely toothed margin; (**Q**) entire leaf margin entire of (**J**) *C. defoliatum* (enlarged image); (**R**) toothed leaf margin of (**H**) *C. faberi* (enlarged image); (**S**) articulate leaf of (**C**) *C. hookerianum* (enlarged image). ap, apex; pe, petioles; lo, lobed; t, toothed; en, entire; pv, primary veins; sv, secondary veins; ar, articulate.

The taxonomy of *Cymbidium* has been problematic at the level of infrageneric categories, including species, sections, and subgenera, due to some overlapping floral morphological features, natural and artificial hybridization, and the discovery of new species [3,12,13,14]. There are some species that can be easily confused based on their morphological features (Figure 1), such as *Cymbidium eburneum* Lindl. (Figure 1A) and *Cymbidium mastersii* Griff. ex Lindl. (Figure 1B), due to both possessing white and showy flowers with long and large leaves, and *Cymbidium qiubeiense* K. M. Feng & H. Li (Figure 1E) and *Cymbidium kanran* Makino (Figure 1I), which share small and greenish flowers with narrow strap leaves [9]. However, these easily confused species should be recognized as separate taxa based on genomic and anatomical data from their chloroplasts [9,15,16,17]. Furthermore, the taxonomy of *Cymbidium* members from China has been controversial; they were divided into three ‘stable’ subgenera, namely subgen. *Cyperorchis*, subgen. *Jensoa*, and subgen. *Cymbidium*, but with each containing different types of sections [9]. Compared with the previous system [3], this one proposed some new sections species whose identification was previously uncertain [9]. For example, in subgen. *Cyperorchis*, *Cymbidium wenshanense* Y. S. Wu & F. Y. Liu was raised to sect. *Annamaea* [9] from sect. *Iridorchis* [3]. However, the categories of subgenus and section were neglected or missed in other classification systems when it came to these confusing differentiations [1,2]. Recently, molecular phylogenetic evidence has provided new insights into the taxonomy of *Cymbidium*, but significant controversy has remained [14]. It was suggested that *Cymbidium* should not be monophyletic and that it could be divided into two or three subgenera using the DNA markers *matK* and nrITS gathered from 34 species [15]. Subsequently, molecular systematic research revealed that there were paraphyletic/polyphyletic relationships among the three subgenera and that sect. *Cymbidium*, sect. *Cyperorchis*, and sect. *Iridorchis* were non-monophyletic by using ITS, *matK*, *trnK*, and 18S-26S sequence data from 37 taxa [16]. Later, it was shown that the species identification of *Cymbidium* was not fully resolved based on DNA barcodes from 237 chloroplast genomes, which came from the sampling of 50 species, indicating that establishing the taxonomy of *Cymbidium* required more evidence and further effort [17].

Leaf morpho-anatomy varies greatly between taxa and has played a key role in the taxonomy and phylogeny of Orchidaceae [2,8]. It was revealed that foliar characteristics, including leaf shape, cross-sectional outline, epidermal cell structure, cuticular thickness, crystal morphology, and vascular bundle arrangement, could be valuable for the identification of orchid species such as in *Bulbophyllum* Thouars [18] and *Dendrobium* Sw. [19]. However, there are limited and scattered data on the leaf morpho-anatomy of some *Cymbidium* species [8,20,21,22], which should be expanded through the collection of more samples. Although other studies have been published on the vegetative anatomy of *Cymbidium*, including analyses of 21 species [22] and 30 species [8], they primarily described leaf anatomic features while lacking detailed illustrations for each species. It was noted that some leaf morphological features varied between species of *Cymbidium* and should receive close attention, including stomatal complex types (tetracytic or cyclocytic–tetracytic, superficial, or depressed), cuticular patterns (smooth or uneven), hypodermal distribution (present or absent adaxially/abaxially), mesophyll organization (homogeneous or heterogeneous), vascular bundle arrangement (collateral or uniseriate), fiber bundle distribution, stegmata presence, and crystal morphology [8,22].

Therefore, the leaf morpho-anatomy of 12 *Cymbidium* species from China (Table 1, Figure 1) was investigated using light microscopy and paraffin sectioning. These species represent three subgenera—subgen. *Cyperorchis* (five species), subgen. *Jensoa* (six species), and subgen. *Cymbidium* (one species)—and include four easily confused species (*Cymbidium mastersii* vs. *C. eburneum*, *C. kanran* vs. *C. qiubeiense*). This study aimed to (1) investigate the leaf morpho-anatomy of these 12 species in detail, including the shape, margin, apex, and the base structure of the leaf epidermal cell, stomatal apparatus, and stigmata; the outline of midrib cross-section and cuticle; the organization of the mesophyll; fiber bundle arrangements; stegmata; and crystal types. Further aims were (2) to select some taxonomic features used for species delimitation based on a comparison of leaf morpho-anatomy and (3) provide a better understanding of the species and taxonomy of *Cymbidium* based on leaf morpho-anatomic evidence, which should be useful for the conservation of Orchidaceae.

## 2. Results

### 2.1. Leaf Morphology

**Figure 2:**
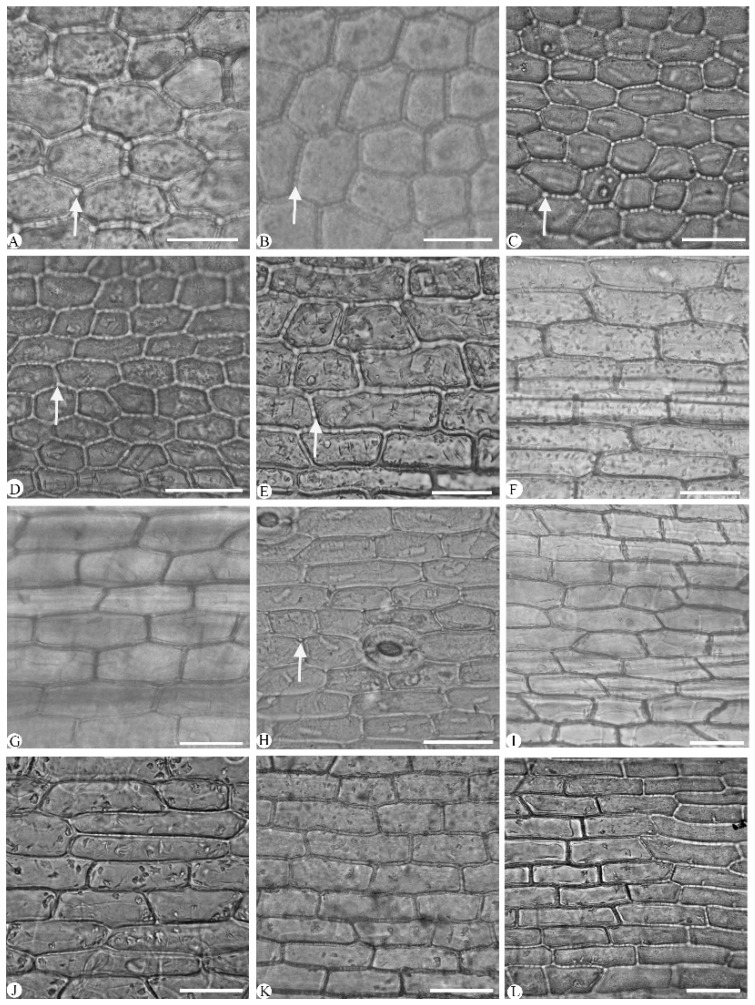
Adaxial leaf epidermis of twelve *Cymbidium* species under light microscope, with epidermal cells and stigmata present (arrow). (**A**–**I**) Polygonal epidermal cells; (**J**–**L**) rectangular epidermal cells. (**A**) *C. lancifolium*; (**B**) *C. floribundum*; (**C**) *C. wenshanense*; (**D**) *C. tracyanum*; (**E**) *C. faberi*; (**F**) *C. eburneum*; (**G**) *C. qiubeiense*; (**H**) *C. mastersii*; (**I**) *C. kanran*; (**J**) *C. hookerianum*; (**K**) *C. defoliatum*; (**L**) *C. serratum*. Scale bars = 50 µm.

**Figure 3:**
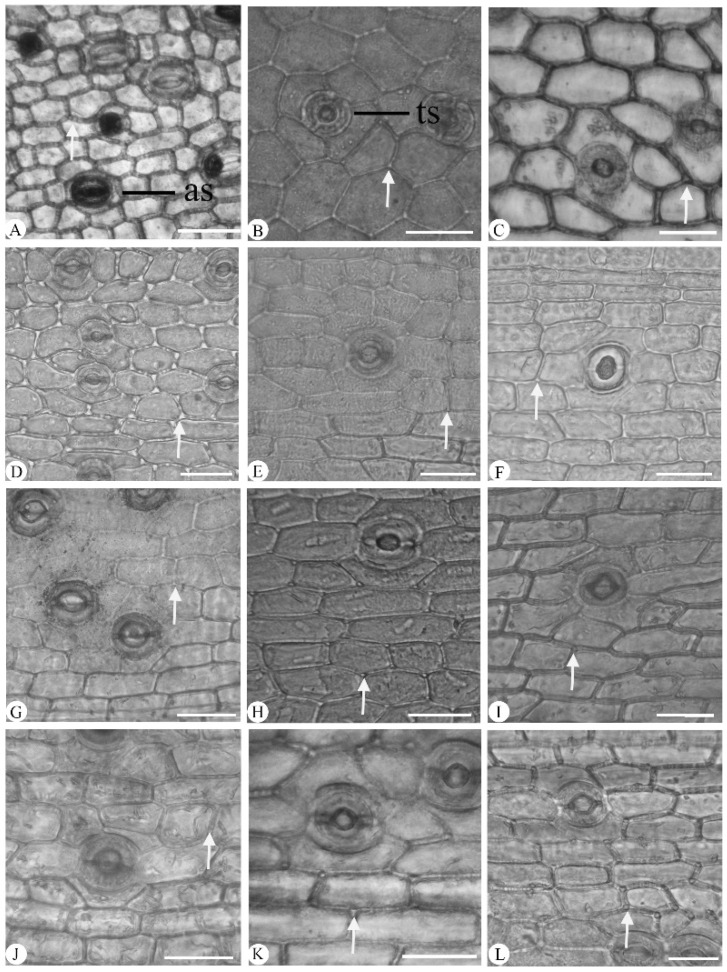
Abaxial leaf epidermis of twelve *Cymbidium* species under light microscope, with epidermal cells, stigmata (arrow), and stomatal shape clearly observable. (**A**–**D**) Broad polygonal epidermal cells; (**E**–**L**) narrow polygonal epidermal cells. (**A**) *C. floribundum*; (**B**) *C. lancifolium*; (**C**) *C. eburneum*; (**D**) *C. wenshanense*; (**E**) *C. kanran*; (**F**) *C. faberi*; (**G**) *C. defoliatum*; (**H**) *C. mastersii*; (**I**) *C. serratum*; (**J**) *C. qiubeiense*; (**K**) *C. hookerianum*; (**L**) *C. tracyanum*. as, anomocytic stomata; ts, tetracytic stomata. Scale bars = 50 μm.

The leaves of the 12 investigated *Cymbidium* species (Figure 1, Table 2) exhibited characteristic monocotyledonous features, displaying predominantly lorate or oblanceolate–oblong shapes with parallel venation patterns. Their vascular architecture consisted of primary and secondary veins extending acropetally from the leaf base to the apex (Figure 1R), consistent with typical monocot leaf morphology. Some leaf morphological features varied between species, including leaf shape, size, margin morphology, apex characteristics, and base structure (the presence or absence of an articulate and petiole). The oblanceolate–oblong leaf shape was observed only in *Cymbidium lancifolium* (Figure 1K), while a lorate leaf was observed in the remaining 11 species (Figure 1A–J,L). Leaf size varied greatly among species. The largest leaf (60–90 cm × 1.3–1.7 cm, L × W) was observed in *C. wenshanense* (Figure 1E), while the smallest leaf (6–17 cm × 4–7 cm, L × W) was that of *C. lancifolium* (Figure 1K). Entire (Figure 1Q) and toothed (Figure 1P) leaf blade margins were seen. Entire leaf margins occurred in seven species, whereas toothed leaf margins were seen in *C. qiubeiense* (Figure 1F), *C. faberi* (Figure 1G), *C. kanran* (Figure 1I), *C. serratum* (Figure 1J), and *C. lancifolium* (Figure 1K). An acute leaf apex was observed in 10 species, while a slightly two-lobed apex (Figure 1O) was seen in *C. eburneum* and *C. mastersii* and a slightly oblique apex (Figure 1M) in *C. floribundum*. Furthermore, an acute leaf apex with a finely toothed apex was found only in *C. lancifolium* (Figure 1P). The leaf base was articulate (Figure 1S) in 10 species, with the exception of *C. faberi* and *C. serratum*. A petiolate (Figure 1N) leaf was exclusively observed in *C. qiubeiense* and *C. lancifolium*, while the remaining 10 species had leaves without petiole (Table 1). In addition, trichomes were absent from both leaf surfaces in 12 species (Figure 1, Figure 2 and Figure 3).

**Figure 4:**
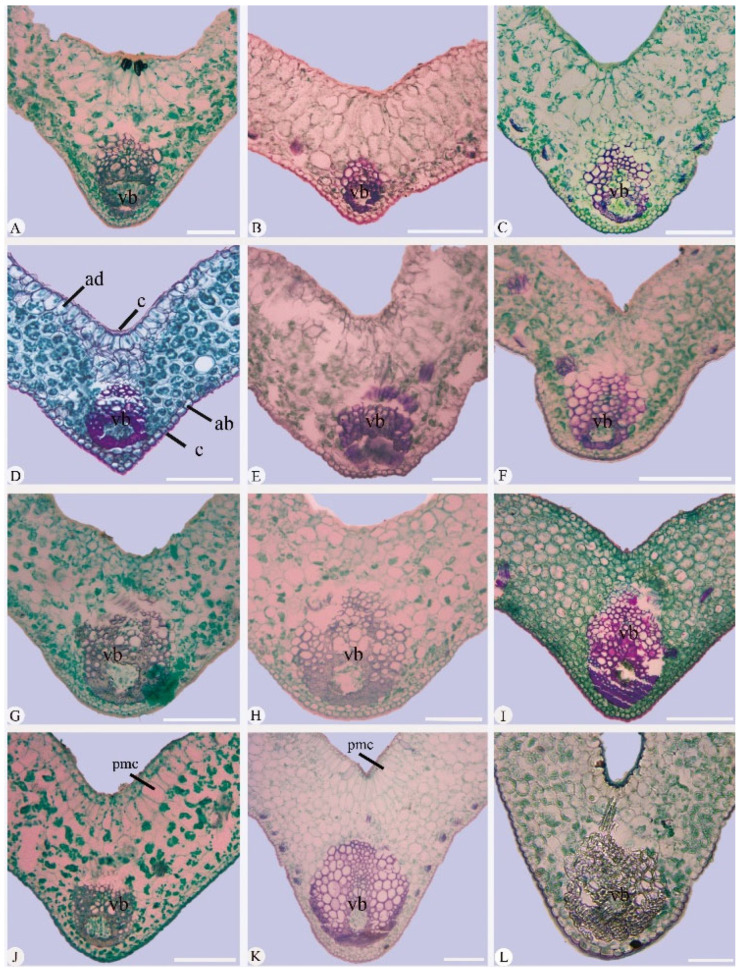
Two types of midrib cross-sections seen in twelve *Cymbidium* species. (**A**–**E**) Shallow V-shape leaf midrib outline; (**F**–**L**) V-shape leaf midrib outline. (**A**) *C. tracyanum*; (**B**) *C. mastersii*; (**C**) *C. wenshanense*; (**D**) *C. serratum*; (**E**) *C. eburneum*; (**F**) *C. kanran*; (**G**) *C. hookerianum*; (**H**) *C. floribundum*; (**I**) *C. faberi*; (**J**) *C. lancifolium*; (**K**) *C. qiubeiense*; (**L**) *C. defoliatum*. ad, adaxial surface; ab, abaxial surface; c, cuticle; vb, vascular bundles; pmc, palisade mesophyll cells. Scale bars = 200 μm.

The epidermal cells were polygonal or rectangular on both leaf surfaces, with straight-arched and significantly thickened anticlinal walls (Figure 2 and Figure 3; Table 3). The adaxial epidermal cells displayed were polygonal in nine species (Figure 2A–I) and rectangular in *Cymbidium hookerianum*, *C. defoliatum*, and *C. serratum* (Figure 2J–L). The abaxial epidermal cells uniformly were polygonal, though variation in their cellular dimensions and proportions was evident. Based on length-to-width (L/W) ratios, these cells were categorized into two distinct types: broad polygonal (1.20–1.65 L/W) and narrow polygonal (1.70–3.40 L/W) (Figure 3, Table 3). This variation is attributed to cellular displacement during stomatal ontogeny (Figure 3 and Figure 4). The broad polygonal cell was found in eight species, mixed with a few rectangular cells (Figure 3E–L), while the narrow polygonal cell was observed in the other four species, which were *C. floribundum* (Figure 3A), *C. lancifolium* (Figure 3B), *C. eburneum* (Figure 3C), and *C. wenshanense* (Figure 3D). The size of the epidermal cells was different in the 12 species (Table 3). The largest epidermal cells were recorded in *C. lancifolium* (62.10 ± 0.87 μm × 59.34 ± 0.90 μm, L × W), while the smallest cells were found in *C. faberi* (23.69 ± 1.11 μm × 13.15 ± 0.48 μm, L × W).

Stigmata were easily observed in the 12 species, consistently appearing along the anticlinal walls of both adaxial and abaxial epidermal cells in a characteristic moniliform arrangement (Figure 2 and Figure 3). Stigmata were universally present in the abaxial epidermal cells of the 12 species (Figure 3); they occurred also on the adaxial surfaces in six species: *Cymbidium lancifolium* (Figure 2A), *C. floribundum* (Figure 2B), *C. wenshanense* (Figure 2C), *C. tracyanum* (Figure 2D), *C. faberi* (Figure 2E), and *C. mastersii* (Figure 2H).

The distribution and morphology of stomatal cells showed significant interspecific variation. All species exhibited abaxial stomata, with the exception of the amphistomatic stomata seen in *Cymbidium mastersii* (Figure 2H). The predominant stomatal type was tetracytic (Figure 3), though occasional anomocytic stomata were identified in *C. floribundum* (Figure 3A). Stomatal complexes were typically surrounded by (4–)5–7 epidermal cells and exhibited two distinct shapes based on their length-to-width (L/W) ratios: elliptic stomata (1.20–1.30 μm, L/W) and round stomata (1.10–1.20 μm, L/W) (Table 3). Elliptic stomata were observed in *C. floribundum* (Figure 3A), *C. lancifolium* (Figure 3B), *C. eburneum* (Figure 3C), *C. faberi* (Figure 3F), *C. mastersii* (Figure 3H), and *C. serratum* (Figure 3I), while round stomata were found in the other six species. Notably, *C. faberi* exhibited sunken stomata (Figure 3F), which was in contrast to the stomatal position observed in the other 11 species.

Our data analysis revealed substantial variation in stomatal dimensions, indices, and densities across the 12 species (Table 3). The largest stomata were observed in *Cymbidium lancifolium* (39.37 ± 0.63 × 29.68 ± 0.56 μm, SL × SW), while the smallest were those in *C. tracyanum* (24.68 ± 0.67 × 20.18 ± 0.39 μm, SL × SW). The stomatal index varied significantly, from 3.98 ± 0.11 μm (*C. serratum*) to 8.01 ± 0.20 μm (*C. eburneum*). Density measurements reached their maximum value in *C. kanran* (1.80 ± 0.15 stomata/μm^2^) and minimum value in *C. lancifolium* (1.10 ± 0.56 stomata/μm^2^).

### 2.2. Leaf Section

*Outline of cross-section of a leaf midrib*: The leaf midrib anatomy of 12 *Cymbidium* species includes a uniseriate epidermis on both the adaxial and abaxial surfaces. The outline of the cross-section of the species’ leaf midrib varied and included V-shape (0–90°) and shallow V-shape (90–180°) types (Figure 4). The shallow V-shape was found in *Cymbidium tracyanum* (Figure 4A), *C. mastersii* (Figure 4B), *C. wenshanense* (Figure 4C), *C. serratum* (Figure 4D), and *C. eburneum* (Figure 4E). In contrast, the V-shaped midrib was characteristic of the remaining seven species, including *C. kanran* (Figure 4F), *C. hookerianum* (Figure 4G), *C. floribundum* (Figure 4H), *C. faberi* (Figure 4I), *C. lancifolium* (Figure 4J), *C. qiubeiense* (Figure 4K), and *C. defoliatum* (Figure 4L). Moreover, the adaxial side was relatively flat in terms of the shape of the cross-section of the leaf midrib in *C. tracyanum*, while the abaxial epidermis displayed a relatively acute angle in *C. serratum* (Figure 4D). The cross-section of the leaf midrib was flat in *C. eburneum*, while it was semicircular in the remaining 11 species.

*Leaf blade thicknesses*: The leaves’ blade thicknesses averaged about 300 μm, with significant variation based on species. The thinnest leaf blade was observed in *Cymbidium kanran* (203.90 ± 1.23 μm) and the thickest in *C. qiubeiense* (490.81 ± 3.60 μm), which is a nearly two-fold difference (Table 4).

*Thickness of the leaf midrib*: The thickness of the leaf midrib averaged 350 μm, with significant variation between species. The midrib thickness ranged from 211.83 ± 3.41 μm in *Cymbidium serratum* to 697.75 ± 18.88 μm in *C. qiubeiense*, which is nearly a three-fold difference. Based on thickness, the midribs could be categorized as either thick (>350 μm) or thin (≤350 μm).

*Mesophyll*: The mesophyll was predominantly homogeneous, consisting of round to elliptical parenchyma cells (Figure 4). Homogeneous mesophyll cells were seen in 10 species, whereas palisade mesophyll cells were present only in *Cymbidium lancifolium* and *C. qiubeiense* (Figure 4J–K). The thickness of the mesophyll averaged 260.43 μm; the largest thickness was found in *C. qiubeiense* (440.36 ± 3.47 μm) and the thinnest in *C. kanran* (177.27 ± 1.10 μm) (Table 4).

*Vascular bundles*: Vascular bundles composed of xylem and phloem were distributed throughout the mesophyll of the 12 species and arranged in a single row at the center of the mesophyll (Figure 4). The size of the vascular bundles varied significantly, with most occupying up to half of the total leaf thickness, except those in *Cymbidium mastersii* (Figure 4B), *C. wenshanense* (Figure 4C), and *C. serratum* (Figure 4D) (Table 4). The thickest vascular bundles averaged 166.09 μm. The smallest vascular bundle was found in *C. serratum* (92.88 ± 3.61 μm) and the largest in *C. qiubeiense* (293.73 ± 22.40 μm), with the thickness of the latter’s vascular bundles up to three times that of the former’s.

**Figure 5:**
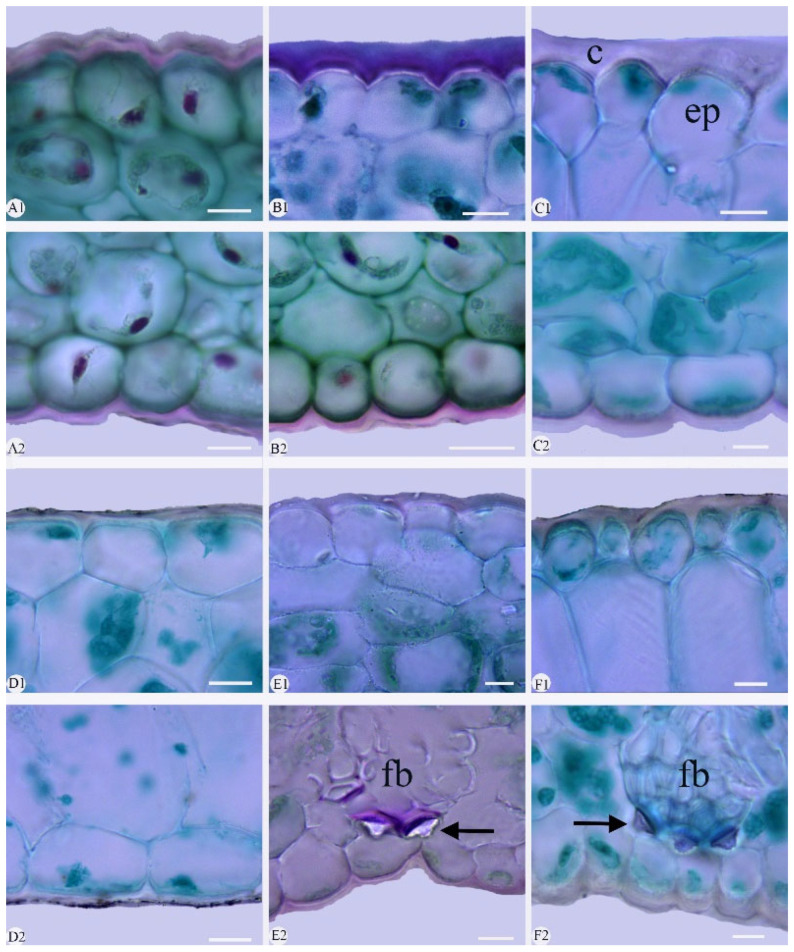
Micro-morphology of cuticle, epidermis, fiber bundle, and stegmata, seen in cross-sections of six *Cymbidium* species. **1**, adaxial surface; **2**, abaxial surface. (**A1**,**A2**) *C. kanran*; (**B1**,**B2**) *C. defoliatum*; (**C1**,**C2**) *C. floribundum*; (**D1**,**D2**) *C. lancifolium*; (**E1**,**E2**) *C. faberi*; (**F1**,**F2**) *C. qiubeiense*. Conical of stegmata = arrow; c, cuticle; ep, epidermis cell; fb, fiber bundles. Scale bars = 10 μm.

**Figure 6:**
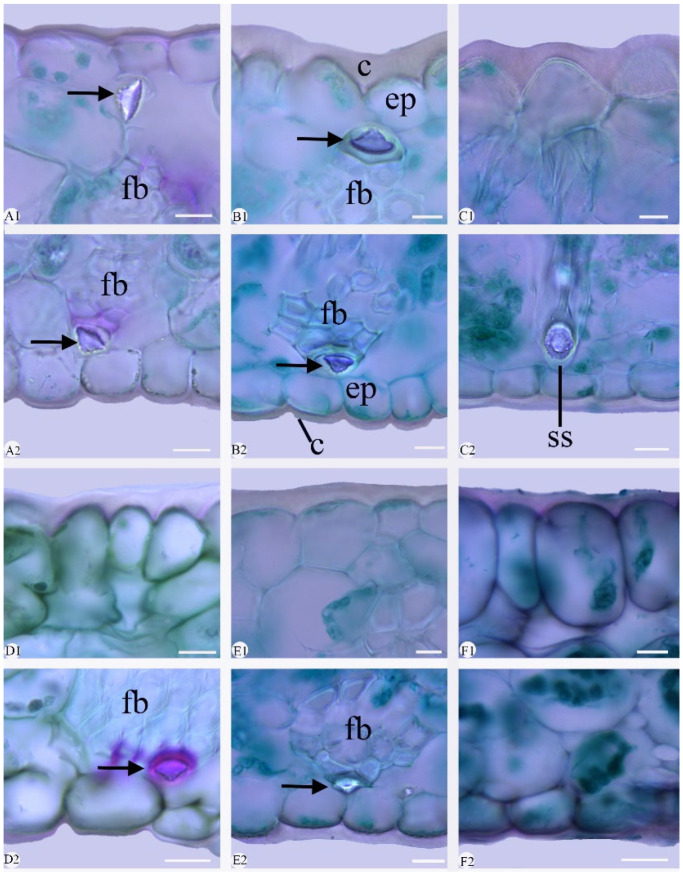
Micro-morphology of cuticle, epidermis, fiber bundle, and stegmata seen in cross-sections of a blade from the other six *Cymbidium* species. **1**, adaxial surface; **2**, abaxial surface. (**A1**,**A2**) *C. mastersii*; (**B1**,**B2**) *C. hookerianum*; (**C1**,**C2**) *C. tracyanum*; (**D1**,**D2**) *C. eburneum*; (**E1**,**E2**) *C. wenshanense*; (**F1**,**F2**) *C. serratum*. Conical of stegmata = arrow. fb, fiber bundles; ss, spherical of stegmata; c, cuticle; ep, epidermis cell. Scale bars = 10 μm.

*Cuticle*: The adaxial cuticle exhibited a greater thickness compared to that of the abaxial surface in the 12 species (Figure 5 and Figure 6). The cuticle was smooth (adaxial and abaxial surface) or corrugated. The cuticle was corrugated in nine species: *C. kanran* (Figure 5A), *C. defoliatum* (Figure 5B), *C. floribundum* (Figure 5C), *C. faberi* (Figure 5E), *C. qiubeiense* (Figure 5F), *C. mastersii* (Figure 6A), *C. hookerianum* (Figure 6B), *C. tracyanum* (Figure 6C), and *C. eburneum* (Figure 6D). The cuticle was smooth in *C. lancifolium* (Figure 5D), *C. wenshanense* (Figure 6E), and *C. serratum* (Figure 6F).

The thicknesses of the cuticles were significantly different between taxa (Figure 5 and Figure 6; Table 4). The thickest cuticle (9.03 ± 0.60 μm) occurred in *C. tracyanum* (Figure 6C), while the thinnest (3.39 ± 0.14 μm) was in *C. mastersii* (Figure 6A). Cuticle thicknesses of up to one-third of the height of the epidermal cells was found in four species, namely *C. defoliatum* (Figure 5B), *C. hookerianum* (Figure 6B), *C. tracyanum* (Figure 6C), and *C. wenshanense* (Figure 6E). A cuticle thickness less than one-third of the height of the epidermal cell occurred in the other eight species: *C. kanran* (Figure 5A), *C. floribundum* (Figure 5C), *C. lancifolium* (Figure 5D), *C. faberi* (Figure 5E), *C. qiubeiense* (Figure 5F), *C. mastersii* (Figure 6A), *C. eburneum* (Figure 6D), and *C. serratum* (Figure 6F).

*Epidermis*: The 12 species possessed a single-layer epidermis on both sides of their leaves (Figure 5 and Figure 6, Table 4). Anatomically, the epidermal cells were of oval (Figure 5A–C,F), polygonal (Figure 5D), and conical (Figure 6B,C); crowded, with small triangular inter-cellular spaces; and sometimes anticlinal or periclinal. Adaxial and abaxial epidermal cells nearly identical in size (1.20–2.00, L/W) were found in six species: *Cymbidium kanran* (Figure 5A), *C. defoliatum* (Figure 5B), *C. floribundum* (Figure 5C), *C. lancifolium* (Figure 5D), *C. faberi* (Figure 5E), and *C. qiubeiense* (Figure 5F). The adaxial epidermal cells were clearly larger than the abaxial epidermal cells (2.01–4.10, L/W) in the other six species: *C. mastersii* (Figure 6A), *C. hookerianum* (Figure 6B), *C. tracyanum* (Figure 6C), *C. eburneum* (Figure 6D), *C. wenshanense* (Figure 6E), and *C. serratum* (Figure 6F). The largest epidermal cells were observed in *C. serratum* (4.02, ET_ad_/ETab) and were approximately four times the size of the abaxial ones.

*Fiber bundle*: Fiber bundles were irregularly distributed within the mesophyll, typically aligned parallel to the vascular bundles and separated from the epidermis by one or two cells (Figure 5 and Figure 6). Two distribution patterns were observed: (1) the fiber bundle was adjacent to both epidermises in *Cymbidium mastersii* (Figure 6A) and *C. hookerianum* (Figure 6B), and (2) the fiber bundle wasrestricted to the abaxial epidermis in *C. faberi* (Figure 5E), *C. qiubeiense* (Figure 5F), *C. eburneum* (Figure 6D), and *C. wenshanense* (Figure 6E). Notably, six species lacked fiber bundles entirely: *C. kanran* (Figure 5A), *C. defoliatum* (Figure 5B), *C. floribundum* (Figure 5C), *C. lancifolium* (Figure 5D), *C. tracyanum* (Figure 6C), and *C. serratum* (Figure 6F).

*Stegmata*: Stegmata, with conical, spherical silica bodies associated with fiber bundles and mesophyll, were observed in seven species, but absent in the other five: *Cymbidium kanran* (Figure 5A), *C. defoliatum* (Figure 5B), *C. floribundum* (Figure 5C), *C. lancifolium* (Figure 5D), and *C. serratum* (Figure 6E). In the former group, conical silica bodies associated with fiber bundles were present in six species: *C*. *faberi* (Figure 5E), *C. qiubeiense* (Figure 5F), *C. mastersii* (Figure 6A), *C. hookerianum* (Figure 6B), *C. eburneum* (Figure 6D), and *C. wenshanense* (Figure 6E). Stegmata with spherical silica bodies immersed in the mesophyll were found only in *C. tracyanum* (Figure 6C).

**Figure 7:**
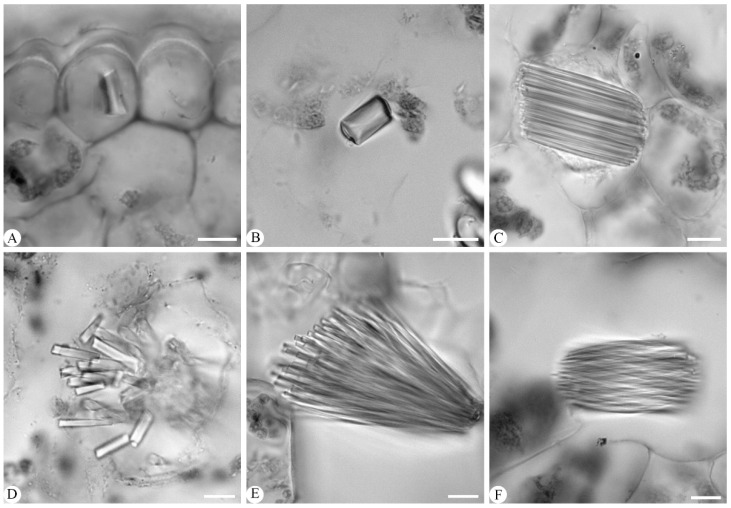
Three types of crystal seen in six *Cymbidium* species. (**A**,**B**) Terete crystal in (**A**) *C. faberi* and (**B**) *C. tracyanum*; (**C**–**E**) long tube crystal bundles in (**C**) *C. qiubeiense*, (**D**) *C. kanran,* and (**E**) *C. mastersii*; and (**F**) raphide crystals in *C. lancifolium*. Scale bars = 10 μm.

*Crystals*: Crystals were observed in six species, but absent in the remaining six. In the six species containing crystals, three crystals were identified, including the terete crystal in *Cymbidium faberi* (Figure 7A) and *C. tracyanum* (Figure 7B); long tube crystal bundles in *C. qiubeiense* (Figure 7C), *C. kanran* (Figure 7D), and *C. mastersii* (Figure 7E); and raphide, which was found exclusively in *C. lancifolium* (Figure 7F). The crystals were primarily distributed within heteromorphic mesophyll cells, except in the epidermal cells of *C. faberi* (Figure 7A).

## 3. Discussion

### 3.1. Taxonomic Significance of Leaf Morpho-Anatomy in Cymbidium

Some data on the leaf morpho-anatomy of some *Cymbidium* species have been reported, but without detailed illustrations [21,22]. Here, the leaf morpho-anatomy of 12 species was investigated thoroughly, although that of five species had previously been reported, including *C. faberi*, *C. lancifolium*, *C. floribundum*, and *C. tracyanum* [21,22]. It was shown that most features of the leaf morpho-anatomy of 12 *Cymbidium* species were consistent with those from other orchids that have been investigated [18,19,23,24,25,26]. However, some features were different between taxa, exhibiting taxonomic significance, including the shape of the epidermal cell, stomata, vascular bundle, cuticle, mesophyll, fiber bundle, and stegmata, as discussed below.

#### 3.1.1. Leaf Epidermal Cell

The leaf epidermal cells were polygonal or rectangular, with straight anticlinal walls. While this is common in Orchidaceae, it varies between taxa [23,24,25,26]. Here, polygonal cells were predominant on both leaf surfaces in nine species, while rectangular cells were restricted to the adaxial surface in *Cymbidium hookerianum*, *C. defoliatum*, and *C. serratum*, and rectangular adaxial epidermal cells were seen in *C. hookerianum* and *C. kanran*. It was confirmed that polygonal epidermal cells were observed on both surfaces of the leaves of *C. lancifolium*, *C. faberi*, *C. floribundum*, and *C. tracyanum* [21,22]. However, both adaxial and abaxial epidermal cells were found to be polygonal in *C. kanran*, which is in contrast to the rectangular epidermal cells previously recorded for the species [22]. In addition, trichome on the leaf epidermis was recorded in *C. eburneum* and *C. lancifolium* [27], but it was not observed here, nor was it in other research [22].

#### 3.1.2. Stomata

Stomata and their distribution vary among orchid taxa, which could be of taxonomic significance [27,28,29,30]. Five types of stomata have been identified in Orchidaceae, including anomocytic, cyclocytic, paracytic, pentacytic, and tetracytic stomata [21,22,27,28]. Among them, tetracytic stomata are predominantly observed in the 23 genera from the tribe Cymbidieae [24]. Here, tetracytic stomata were observed in 11 species, while anomocytic stomata were seen only in *Cymbidium floribundum*. Tetracytic stomata were also recorded in six other *Cymbidium* species: *Cymbidium ensifolium* (L.) Sw., *C. aloifolium* (L.) Sw. [29], *C. cyperifolium* Wall. ex Lindl., *C. goeringii* (Rchb. f.) Rchb. F., *C. devonianum* Paxton, and C. *bicolor* Lindl. [22]. Meanwhile, abaxial stomata were observed in 11 species, while amphistomatic stomata were found only in C. *mastersii*. Amphistomatic stomata were previously recorded in *Cymbidium canaliculatum* R.Br. [22]. Therefore, it was proven that tetracytic stomata are more common than anomocytic stomata in *Cymbidium* [22,28]. Similarly, it was suggested that abaxial stomata were more predominant than anomocytic stomata in *Cymbidium*. In addition, anomocytic stomata were identified in *C. floribundum*, which is in contrast to the tetracytic type previously described [22].

#### 3.1.3. Vascular Bundles

The amphivasal vascular bundles, composed of xylem and phloem, were distributed throughout the mesophyll and arranged in a single row at the center of the mesophyll in the 12 species observed, which was consistent with the vascular bundles of other *Cymbidium* species [22]. However, both amphivasal and bicollateral vascular bundles were recorded in *Dendrobium* [19]. Vascular bundles were evident and accounted for approximately half of the total leaf blade thickness in nine species, but not in *Cymbidium mastersii*, *C. wenshanense*, and *C. serratum*. This feature was also noted in *C. ensifolium*, *C. goeringii*, and *C. sinense* (Jack. ex Andr.) Willd. [21].

#### 3.1.4. Cuticle

Meanwhile, the shape and thickness of the cuticle varied between orchid species [31]. Commonly, plant cuticles play an important role in the interaction between plants and their environment, reducing the solar radiation absorbed and the temperature of the plant by reflecting sunlight and reducing transpiration [32]. Here, a corrugated cuticle was observed in nine species, whereas a smooth cuticle was seen in *C. lancifolium*, *C. wenshanense*, and *C. serratum*. Notably, corrugated cuticles were found in *C. kanran*, *C. floribundum*, and *C. hookerianum*, which is in contrast to the smooth cuticle documented in previous data [22]. In addition, the thickness of the cuticle varied between species, which was not mentioned in previous research [22]. A thick cuticle, observed to reach up to one-third of the epidermal cell height, was observed in four species *(C. defoliatum*, *C. hookerianum*, *C. wenshanense*, and *C. tracyanum*), while thin cuticles were observed in the other eight species studied.

#### 3.1.5. Mesophyll

The mesophyll cells are often homogeneous but occasionally heterogeneous in Orchidaceae [21,22,23]. Here, the mesophyll was homogeneous in 10 *Cymbidium* species, but heterogeneous, with a pronounced palisade mesophyll cell, in *C. qiubeiense* and *C. lancifolium*. It was noted that the latter two species were distinguished from the other 10 by the presence of a petiole. It seemed that the differentiation of the heterogenous mesophyll might be related to the presence of a petiole in Cymbidium. A heterogeneous mesophyll with a likely palisade layer was also recorded in 3 out of 21 species *Cymbidium* elsewhere (*C. aloifolium*, *C. bicolor*, and *C. finlaysonianum* Lindl.), which was presumed to be the result of evolution for adaption to high-light-level habitats, since members of *Cymbidium* grow on isolated trees in tropical lowland forests or on rocks [22]. However, heterogeneous mesophyll is not present in other epiphytic orchids, such as *Bulbophllyum* [18] and *Dendrobium* [19].

#### 3.1.6. Fiber Bundle

The presence and position of fiber bundles varied between the species of *Cymbidium* [21,22,24]. Here, fiber bundles occurred in six species but were absent in the other six. In the former group, fiber bundles adjacent to the ad- and abaxial epidermises was observed in *C. mastersii* and *C. hookerianum*, while they were restricted to the abaxial epidermis in *C. faberi*, *C. qiubeiense*, *C. eburneum*, and *C. wenshanense*. It was reconfirmed that fiber bundles occurring on both surfaces were previously recorded in *C. floribundum*, *C. tracyanum*, *C. hookerianum*, and *C. kanran*, but thought to be absent in *C. lancifolium* [22]. However, fiber bundles were clearly absent in *C. floribundum* and *C. tracyanum* in this research but described as present, without illustration, in older data [22]. Interestingly, fiber bundles were observed to be very numerous on the abaxial surface but completely absent on the adaxial surface in *C. ensifolium* [29]. It was revealed that fiber bundles were absent in *Bulbophyllum* [18]. However, the state of the fiber bundles varies between taxa at the levels of genus and subtribe in Orchidaceae. For example, fiber bundles are absent in *Dendrobium* section *Stachyobium* [19] but present in *Dendrobium arachnoideum* Schltr. and *D. guttulatum* Schltr [31]. And fiber bundles have been observed in some members of Oncidiinae (Maxillarieae), such as *Ionopsis utricularioides* (Sw.) Lindl., *Aspasia lunata* Lindl., and *Oncidium boothianum* Rchb.f., although they are notably absent in *Erycina echinata* (Kunth) Lindl. [33].

#### 3.1.7. Stegmata

The state, type, and position of the stegmata are different in different Orchidaceae [22,34,35]. The shape of the stegmata varied, but included spherical, conical, and rough-surfaced silica bodies associated with vascular bundles and fiber bundles [22,24,34]. Here, stegmata with conical and spherical silica bodies that were associated with fiber bundles and mesophyll were observed in seven species, but absent in the other five species (*C. kanran*, *C. defoliatum*, *C. floribundum*, *C. lancifolium*, and *C. serratum*). The stegmata associated with fiber bundles were common in six species, but those with spherical silica bodies immersed in the mesophyll were found only in *Cymbidium tracyanum*. In addition, stegmata were evidently absent from *C. kanran* and *C. lancifolium*, although they were previously recorded as present, without illustrations, in previous data [22].

#### 3.1.8. Crystals

Crystals such as raphide crystals, spherical silica body crystals, and prismatic crystals are common in leaf anatomy and vary in shape and size among orchid taxa [19,36]. Here, crystals were present in six species, but absent in the other six. The crystals were mainly observed in heteromorphic mesophyll cells, except in *Cymbidium faberi*, where they were found in epidermal cells. In the former cells, three types of crystals were identified: terete crystals, long tube crystal bundles, and raphide crystals. Terete crystals were present in *C. faberi* and *C. tracyanum* and long tube crystal bundles in *C. qiubeiense*, *C. kanran*, and *C. mastersii*. Raphide crystals were only found in *C. lancifolium*, while they have been recorded in *C. ensifolium* and *C. aloifolium* [29]. The crystals varied between orchid taxa. In *Dendrobium* section *Stachyobium*, spherical silica body crystals were found in *D. sinominutiflorum* but prismatic crystals were found in *D. compactum*, *D. monticola*, *D. porphyrochilum*, and *D. strongylanthum*. Crystals such as raphide bundles and druse were observed in some species but absent in four species from *Dendrobium* sect. *Rhizobium* [37]. Three crystals were varied and co-occurring among six species of *Phalaenopsis* [38], including prismatic crystals, spherical silica bodies, and raphide.

#### 3.1.9. Stigmata

Stigmata were evidently observed along the anticlinal wall of polygonal and rectangular epidermal cells in the 12 *Cymbidium* species studied, but they were either not noticed or their taxonomic implications were neglected in previous research [21,22]. Stigmata were predominantly observed on the abaxial epidermis in the 12 *Cymbidium* species, and they were also detected on both the adaxial and abaxial epidermal surfaces in 6 species (*Cymbidium lancifolium*, *C. floribundum*, *C. wenshanense*, *C. tracyanum*, *C. faberi*, and *C. mastersii*). Stigmata were previously observed and noted in *Dendrobium kingianum* Bidwill ex Lindl. and *Bulbophyllum mentosum* Barb. Rodr [18], but without further investigation into this uncommon feature. It was clearly observed, with detailed illustrations, that stigmata were totally absent in five *Dendrobium* species [19] and six *Phalaenopsis* species [38] by the same authors, implying that they paid more attention to their taxonomic significance with larger numbers of samples.

### 3.2. Implications for Infrageneric Taxonomy for Cymbidium

Three subgenera were recognized, subgen. *Cymbidium*, *Cyperorchis*, and *Jensoa*, based on their pollinium number and labellum–column fusion characteristics [11]. This classification was widely adopted by subsequent taxonomists [1,2,9]. However, the delimitation of the sections and species within these subgenera has been unclear when using previously gathered floral morphological and molecular data, meaning more evidence is needed to clarify the delimitation of taxa in *Cymbidium* [1,9]. The leaf morpho-anatomic features of 12 species representing the three subgenera of *Cymbidium* varied greatly, which means they could be used to make taxonomic distinctions as follows below.

#### 3.2.1. Delimitation of Species in subgen. *Cyperorchis*

Five species from subgen. *Cyperorchis* were investigated, including two species (*Cymbidium eburneum* and *C. mastersii*) in sect. *Eburnea* and three (*C. hookerianum*, *C. tracyanum*, and *C. wenshanense*) from sect. *Iridorchis* [3]. Here, it was shown that the shape of the leaf blade tip varied between the two sections; a lorate leaf with a slightly two-lobed tip was common in sect. *Eburnea*, compared to the complete tip seen in sect. *Iridorchis*.

Furthermore, *Cymbidium wenshanense* was moved out of sect. *Iridorchis* and transferred into sect. *Annamaea* based on some subtle floral features [9]. It was shown that *C. wenshanense* could be distinguished from the other two species from sect. *Iridorchis* by its large leaf, smooth cuticle, the fiber bundles on its abaxial epidermis, and its lack of crystals. In contrast, *C. hookerianum* and *C. tracyanum* (sect. *Iridorchis*) shared some common features, such as a moderate leaf size and corrugated cuticles, but each of them could be discriminated between by some leaf morpho-anatomic features. *Cymbidium hookerianum* was characterized by polygonal adaxial epidermal cells, fiber bundles on both epidermal surfaces, and conical silica bodies, whereas *C. tracyanum* featured polygonal adaxial and abaxial epidermal cells and lacked fiber bundles and spherical silica bodies.

*Cymbidium mastersii* and *C. eburneum* were placed in sect. *Eburnea*, which seemed to contain species that were easily confused due to their overlapping morphological features, such as the size and the shape of the plant, leaf, and flowers. However, each of them was a separate species, since they formed sister clades based on their molecular data [2,3]. Each of them could be recognized by their comparative leaf morpho-anatomy. *Cymbidium mastersii* is characterized by the presence of stigmata on both surfaces, hypostomatous stomata, fiber bundles near both epidermis layers, the thinnest cuticle, and long tube crystal bundles. In contrast, *C. eburneum* features amphistomatic stomata, the highest stomatal index, stigmata on its abaxial surface, and fiber bundles confined to the abaxial epidermis, while lacking these crystals.

#### 3.2.2. Species Delimitation in subgen. *Jensoa*

Five species were taken from subgen. *Jensoa*, including *Cymbidium defoliatum*, *C. qiubeiense*, *C. kanran*, *C. faberi*, and *C. serratum*, from sect. *Jensoa* and *C. lancifolium* from sect. *Geocymbidium* [3]. It was proposed that *C. faberi* and *C. serratum* should be moved from sect. *Jensoa* to sect. *Nanula*, considering their lack of an articulate leaf base [9]. However, no clear evidence from leaf morpho-anatomy could be used for the delimitation of these two sections. There were some morpho-anatomic features that could easily be used for a clear discrimination of sect. *Geocymbidium* from the other two sections, such as the maximal stomatal size, minimal stomatal density, and an articulate leaf shape, among the 12 species studied. Of course, it seemed reasonable to place *C. lancifolium* in the monotypic sect. *Geocymbidium*, because it was distinguished by an oblanceolate–oblong leaf with a petiole. In addition, leaves with petioles and heterogenous mesophyll were not seen in most *Cymbidium* members, but present in *C. lancifolium* and *C. qiubeiense*, which could be used as a diagnostic feature for them.

And then there exist two species which are easily confused, namely *Cymbidium faberi* and *C. serratum*, due to the similar morphology of their plants, leaves, and flowers [3,9]. However, it was possible to separate them using molecular evidence [2] and leaf morpho-anatomic features in this research. *Cymbidium faberi* was characterized by a smaller epidermal cell, stigmata on both surfaces, sunken stomata, a V-shaped midrib, fiber bundles restricted to the abaxial epidermis, and the presence of crystals. In contrast, *C. serratum* possessed a larger epidermal cell, stigmata on the adaxial epidermis, flat stomata, a shallow V-shape midrib cross-section, and an absence of both fiber bundles and crystals.

In addition, there are three species which share some common features and are easily confused: *Cymbidium defoliatum*, *C. qiubeiense* and *C. kanran* [3,9]. Each of them could be identified using molecular data [2], and also by their leaf morphology, including the leaf blade margin (entire or toothed) and the state of their petiole. *C. defoliatum* was characterized by an entire leaf margin, while the latter two had toothed leaf margins. *C. qiubeiense* was characterized by a petiolate leaf with heterogenous mesophyll, a thicker leaf blade, larger vascular bundles, and the presence of stegmata and crystals. Conversely, *C. defoliatum* and *C. kanran* were distinguished by a leaf without a petiole and with homogenous mesophyll, smaller vascular bundles, and the absence of both stegmata and crystals.

#### 3.2.3. Species Delimitation in subgen. *Cymbidium*

There was only one species (*Cymbidium floribundum*) sampled from Subgen. *Cymbidium*, which should be investigated further using more samples. *Cymbidium floribundum* was greatly distinguished from the other 11 species by having the only anomocytic stomata, which have rarely been observed or noticed in the other orchid data available.

#### 3.2.4. Species Delimitation of Cymbidium Based on Leaf Morpho-Anatomic Features

It was shown that some leaf morpho-anatomic features varied between taxa and could be used for species delimitation in *Cymbidium*. Accordingly, a key to the 12 *Cymbidium* species observed was proposed based on their leaf morpho-anatomic features (Appendix A). This would provide a better understanding of the species delimitation and taxonomy of *Cymbidium*.

## 4. Materials and Methods

*Taxa selected*: Twelve species of *Cymbidium* from China (Table 1 and Table 2, Figure 1) were selected, representing three subgenera and five or seven sections, based on previous taxonomic references [3,9]. Six species were from the subgenera *Jensoa* and covered its two sections (*Cymbidium defoliatum* Y. S. Wu & S. C. Chen; *C. faberi* Rolfe; *C. kanran* Makino; *C. qiubeiense* K. M. Feng & H. L; and *C. serratum* Schltr. *from* sect. *Jensoa* and *C. lancifolium* from sect. *Geocymbidium* Hook). Five species were from subgen. *Cyperorchis* (*C. hookerianum* Hook., *C. tracyanum* L. Castle and *C. wenshanense* Y. S. Wu & F. Y. Liu from sect. *Iridorchis*; *C. eburneum* Lindl. and *C. mastersii* Griff. ex Lindl. from sect. *Eburnea*), and one species (*C. floribundum* Lindl.) was from subgen. *Cymbidium,* according to previous taxonomic data [3].

*Sample preparation*: Samples from plants of these 12 *Cymbidium* species were prepared as follows. Plants of these 12 *Cymbidium* species have been cultivated and have bloomed every year for nearly twenty years in the Kunming Institute of Botany, Chinese Academy of Sciences, and in the Orchid Germplasm Resource Nursery, Yunnan Fengchunfang Biotechnology Company Limited, Fumin, Yunnan, China (25° 20′ 01″ N, 102° 27′ 26″ E). Three to five mature leaves from several plants per species were cut into pieces, fixed in FAA (50% alcohol/acetic acid/formaldehyde = 90:5:5) [39], and preserved in 50% alcohol (Tianjin Zhiyuan Chemical Reagent Co., Ltd., Tianjin, China). The length and width of the fully expanded leaves were measured in the field with the help of a glass scale ruler and graph paper.

*Observation of leaf morpho-anatomic features*: The epidermis was disassociated using the method described by Sun and Jiang [40]. Leaf epidermal characteristics were manually recorded from leaf edge fragments measuring approximately 1 cm × 1 cm, which were immersed in 30% H_2_O_2_–CH_2_COOH (Xilong Scientific Co., Ltd., Guangzhou, China) at 60 °C for 12–36 h and stained with 1% safranin for 2–3 min. Transversal sections (0.5 cm × 0.5 cm) from between the mid-vein and leaf edge were dehydrated, embedded in paraffin wax (melting point = 57 °C), sectioned into 8–30 μm thicknesses using a manual rotary microtome (Leica RM225RTS, Wetzlar, Germany), and stained with 1% safranin and 1% fast green (HeFei BoMei Biotechnology Co., Ltd., HeFei, China). The samples were mounted on permanent slides using Canada balsam (Beijing Labgic Technology Co., Ltd., Beijing, China) and observed and photographed using a Nikon E100 light microscope (Nikon, Tokyo, Japan). The terminology used for the stomatal complex types followed that of Patel [41]. Thirty measurements were taken for each documented value—the lengths and widths of adaxial and abaxial leaf epidermal cells, stomata (index and density), leaf thickness, midrib thickness, mesophyll thickness, vascular bundle thickness (measuring only the thickness of the phloem and xylem of midvein vascular bundles), and epidermis and cuticle thicknesses of both adaxial and abaxial surfaces—using ImageJ software (https://imagej.nih.gov/ij/download.html) (NIH, Bethesda, MD, USA). Stomatal index = number of stomata/(number of stomata + number of ordinary epidermal cells) × 100%.

*Data Analysis*: Statistical analyses were performed using Excel 2016 (Microsoft, Redmond, WA, USA), and means and standard deviations were calculated (mean ± SD) while retaining two decimal places. To evaluate significant differences in morphoanatomical features among the 12 species of *Cymbidium*, the data were tested separately via a one-way analysis of variance (ANOVA) using SPSS 25.0 software (IBM, Armonk, NY, USA). The means were separated using Duncan’s multiple range test, and differences between the mean were considered significant at a *p* < 0.05.

## 5. Conclusions

The leaf morpho-anatomy of 12 *Cymbidium* species was investigated using a microscope and paraffin sections. Their comparative leaf morpho-anatomy indicated that some leaf morpho-anatomical features of the 12 *Cymbidium* species varied between species, including the leaf epidermal cell, stomata, mesophyll, fiber bundle, stegmata, and type of crystal present. These features could be used for species identification. Accordingly, a taxonomic key to the 12 *Cymbidium* species from China was proposed based on these leaf morpho-anatomical features. However, the taxonomic implications of these aspects of leaf morpho-anatomy for different sections and subgenera of *Cymbidium* could not be definitely proposed since the data available were limited. Therefore, further investigation with extended sampling should be conducted, which would provide a better understanding of the taxonomy of *Cymbidium*.

## Figures and Tables

**Table 1:** Taxonomic data of twelve *Cymbidium* species investigated.

Species	Chen et al., (1999) [3]	Liu et al., (2006) [9]	Distribution	Voucher
——	Subgen. *Cyperorchis*	Subgen. *Cyperorchis*	——	——
*C. eburneum*	Sect. *Eburnea*	Sect. *Eburnea*	China, India, Myanmar, Nepal, Vietnam	Lilu20190228
*C. mastersii*	Sect. *Eburnea*	Sect. *Eburnea*	Asia	Lilu20220725
*C. hookerianum*	Sect. *Iridorchis*	Sect. *Iridorchis*	China, India, Nepal, Vietnam	Lilu20200526
*C. tracyanum*	Sect. *Iridorchis*	Sect. *Iridorchis*	China, Myanmar, Thailand	Lilu20181218
*C. wenshanense*	Sect. *Iridorchis*	Sect. *Annamaea*	China, Vietnam	Lilu20200324
——	Subgen. *Jensoa*	Subgen. *Jensoa*	——	——
*C. defoliatum* *	Sect. *Jensoa*	Sect. *Jensoa*	China	Lilu20201112
*C. kanran*	Sect. *Jensoa*	Sect. *Jensoa*	China, Japan, Korea	Lilu20201111
*C. qiubeiense* *	Sect. *Jensoa*	Sect. *Jensoa*	China	Lilu20220114
*C. faberi*	Sect. *Jensoa*	Sect. *Nanula*	China, India, Nepal	Lilu20170725
*C. serratum* *	Sect. *Jensoa*	Sect. *Nanula*	China	Lilu20220715
*C. lancifolium*	Sect. *Geocymbidium*	Sect. *Geocymbidium*	Asia, Oceania	Lilu20180728
——	Subgen. *Cymbidium*	Subgen. *Cymbidium*	——	——
*C. floribundum*	Sect. *Floribunda*	Sect. *Floribunda*	China, Vietnam	Lilu20180730

* Chinese endemic species.

**Table 2:** Leaf morphology of twelve *Cymbidium* species.

Species	Shapes	Size (L × W), cm	Margin	Apex	Base	Petiole
*C. defoliatum*	lorate	10–40 × 0.5–1	entire	acute	articulate	absent
*C. eburneum*	lorate	57–65 × 1.4–2.1	entire	acute, slightly two lobed	articulate	absent
*C. faberi*	lorate	25–80 × 0.4–1.2	toothed	acute	absent	absent
*C. floribundum*	lorate	22–50 × 0.8–1.8	entire	acute, slightly oblique	articulate	absent
*C. hookerianum*	lorate	35–60 × 1.4–2.3	entire	acute	articulate	absent
*C. kanran*	lorate	40–70 × 0.9–1.7	toothed	acute	articulate	absent
*C. lancifolium*	oblanceolate oblong	6–17 × 4–7	toothed	acute	articulate	petiole
*C. mastersii*	lorate	24–75 × 1.1–2.5	entire	acute, slightly two lobed	articulate	absent
*C. qiubeiense*	lorate	30–80 × 5–10	toothed	acute	articulate	petiole
*C. serratum*	lorate	20–40 × 0.5–0.9	toothed	acute	absent	absent
*C. tracyanum*	lorate	55–80 × 1.5–3.4	entire	acute	articulate	absent
*C. wenshanense*	lorate	60–90 × 1.3–1.7	entire	acute	articulate	absent

**Table 3:** Leaf epidermal features of twelve *Cymbidium* species.

Species	Adaxial Epidermis			Abaxial Epidermis							
	EL_ad_	EW_ad_	L/W	EL_ab_	EW_ab_	L/W	SL	SW	SL/SW	SI	SD
*C. defoliatum*	45.48 ± 2.11 e	17.35 ± 0.40 e	2.62	42.85 ± 1.71 bc	18.17 ± 0.54 bc	2.36	32.49 ± 0.63 a	27.70 ± 0.37 ab	1.17	5.21 ± 0.23 d	1.37 ± 0.10 bcd
*C. eburneum*	56.32 ± 3.42 abc	19.49 ± 0.47 d	2.89	25.91 ± 1.25 f	16.13 ± 0.24 cdef	1.60	31.71 ± 0.56 bcd	25.99 ± 0.60 a	1.22	8.01 ± 0.20 a	1.67 ± 0.13 ab
*C. faberi*	58.54 ± 2.06 ab	13.26 ± 0.59 f	4.41	45.61 ± 2.10 ab	16.80 ± 0.72 cde	2.71	30.65 ± 0.55 cd	24.41 ± 0.37 a	1.25	6.87 ± 0.29 bc	1.27 ± 0.82 cd
*C. floribundum*	23.69 ± 1.11 g	13.15 ± 0.48 f	1.80	20.81 ± 0.73 g	17.14 ± 0.50 cde	1.21	24.68 ± 0.67 f	20.18 ± 0.39 d	1.22	6.37 ± 0.21 c	1.47 ± 0.10 bcd
*C. hookerianum*	54.02 ± 1.80 bc	15.00 ± 1.10 f	3.60	31.72 ± 1.10 e	13.67 ± 0.60 de	2.32	32.33 ± 0.63 bc	27.54 ± 0.62 a	1.18	6.60 ± 0.24 bc	1.36 ± 0.10 bcd
*C. kanran*	52.30 ± 2.14 bcd	17.92 ± 0.33 de	2.92	36.64 ± 1.40 d	16.71 ± 0.67 cde	2.19	30.19 ± 0.61 cd	26.22 ± 0.42 ab	1.15	6.79 ± 0.30 bc	1.80 ± 0.15 a
*C. lancifolium*	62.10 ± 0.87 a	59.34 ± 0.90 a	1.05	38.59 ± 2.06 cd	26.65 ± 0.68 a	1.45	39.37 ± 0.63 f	29.68 ± 0.56 d	1.33	4.19 ± 0.21 e	1.10 ± 0.56 d
*C. mastersii*	50.73 ± 2.50 cde	22.91 ± 0.45 c	2.21	40.81 ± 2.34 cd	16.93 ± 0.56 cde	2.41	33.34 ± 0.48 ab	27.24 ± 0.69 a	1.25	4.00 ± 0.20 e	1.36 ± 0.10 bcd
*C. qiubeiense*	47.54 ± 1.80 de	14.12 ± 0.46 f	3.36	30.10 ± 0.73 f	15.87 ± 0.38 e	1.89	29.50 ± 0.53 d	25.19 ± 0.65 c	1.17	6.38 ± 0.20 c	1.37 ± 0.10 bcd
*C. serratum*	56.08 ± 2.92 abc	13.43 ± 0.50 f	4.17	48.17 ± 2.34 a	14.59 ± 0.33 fg	3.30	30.33 ± 0.47 d	24.14 ± 0.45 c	1.27	3.98 ± 0.11 e	1.30 ± 0.98 cd
*C. tracyanum*	27.98 ± 1.40 g	14.10 ± 0.49 f	1.98	25.65 ± 0.43 f	13.68 ± 0.54 b	1.89	26.60 ± 0.53 e	24.53 ± 0.52 c	1.08	6.45 ± 0.21 c	1.50 ± 0.12 abc
*C. wenshanense*	34.38 ± 1.04 f	26.30 ± 0.75 b	1.31	22.40 ± 2.08 cd	17.78 ± 0.63 cd	1.25	30.15 ± 0.36 d	27.61 ± 0.48 a	1.09	7.23 ± 0.21 b	1.50 ± 0.10 abc

Mean ± SE (n = 30). Different letters in the same line indicate statistical differences *p* < 0.05 (ANOVA). EL_ad_, length of adaxial ordinary epidermal cell; W_ad_, width of adaxial ordinary epidermal cell; EL_ab_, length of abaxial ordinary epidermal cell; EW_ab_, width of abaxial ordinary epidermal cell; SL, length of stomata; SW, stomata width; SI, stomatal index; SD, stomatal density.

**Table 4:** Leaf anatomic features of twelve *Cymbidium* species.

Species	LT	TM	MT	VBT	ET_ad_	ET_ab_	ET_ad_/ET_ab_	C_ad_	C_ab_
*C.lancifolium*	317.71 ± 6.73 c	371.92 ± 3.32 d	268.93 ± 4.09 de	141.47 ± 3.66 ef	27.56 ± 0.61 a	17.68 ± 0.37c	1.58	5.65 ± 0.31 cde	3.99 ± 0.21 bc
*C. serratum*	217.16 ± 4.69 f	211.83 ± 3.41 fg	185.63 ± 7.60 h	88.98 ± 7.12 g	44.70 ± 2.00 bc	11.10 ± 0.79 b	4.02	4.14 ± 0.29 fg	1.85 ± 0.09 g
*C. mastersii*	215.24 ± 2.67 f	273.61 ± 1.43 f	183.55 ± 2.23 h	92.88 ± 3.61 g	13.77 ± 1.06 bcd	11.45 ± 0.33 b	1.07	3.39 ± 0.14 g	2.19 ± 0.08 fg
*C. kanran*	203.90 ± 1.23 g	239.62 ± 10.58 g	177.27 ± 1.10 h	119.83 ± 4.86 fg	18.01 ± 0.62 f	10.13 ± 0.40 b	1.79	3.54 ± 0.13 g	1.88 ± 0.07 g
*C. Wenshanense*	344.64 ± 7.47 b	423.01 ± 12.86 bc	277.75 ± 3.74 cd	158.16 ± 10.79 de	23.75 ± 0.85 b	10.89 ± 0.20 a	2.18	7.61 ± 0.35 b	4.31 ± 0.21 a
*C. hookerianum*	293.52 ± 3.29 d	325.29 ± 13.44 f	254.94 ± 1.86 f	180.50 ± 2.23 g	16.57 ± 0.52 h	9.13 ± 0.60 de	1.81	7.48 ± 0.41 b	3.75 ± 0.19 c
*C. tracyanum*	245.87 ± 2.66 e	454.14 ± 16.41 b	207.54 ± 2.21 g	126.77 ± 4.90 ef	22.14 ± 1.21 cdef	10.82 ± 0.91 c	2.05	9.03 ± 0.60 a	2.34 ± 0.12 ef
*C. faberi*	347.06 ± 3.14 b	449.16 ± 10.23 b	287.52 ± 2.34 c	250.86 ± 15.87 b	21.00 ± 0.63 b cde	14.04 ± 0.39 c	1.54	4.93 ± 0.17 ef	3.64 ± 0.10 c
*C. eburneum*	293.45 ± 4.68 d	312.34 ± 4.56 e	253.19 ± 3.53 f	150.82 ± 4.91 ef	23.43 ± 0.89 b	10.26 ± 0.29 a	2.28	5.21 ± 0.29 cde	2.65 ± 0.12 de
*C. defoliatum*	344.26 ± 2.26 b	410.61 ± 14.31 c	313.80 ± 3.07 b	214.75 ± 15.32 c	15.00 ± 0.33 g	9.76 ± 0.41 c	1.58	5.94 ± 0.19 cd	4.31 ± 0.16 ab
*C. qiubeiense*	490.81 ± 3.60 a	697.75 ± 18.88 a	440.36 ± 3.47 a	293.73 ± 22.40 a	28.18 ± 0.68 ef	12.65 ± 0.32 cd	2.23	6.18 ± 0.35 c	4.51 ± 0.26 ab
*C. floribundum*	302.90 ± 5.14 d	421.45 ± 6.10 bc	262.67 ± 4.39 ef	174.31 ± 6.50 d	20.26 ± 0.36 def	16.33 ± 0.50 e	1.22	4.84 ± 0.18 ef	2.87 ± 0.12 d

Mean ± SE (n = 30). Different letters in the same line indicate statistical differences *p* < 0.05 (ANOVA). LT, leaf thickness; TM, thickness of midrib; MT, mesophyll thickness; VBT, vascular bundle thickness; ETad, thickness of adaxial epidermis; ETab, thickness of abaxial epidermis; Cad, adaxial cuticle thickness; Cab, abaxial cuticle thickness.

## Data Availability

The data that support the findings of this study are available from the corresponding author upon reasonable request.

## Appendix A. A Key to Twelve Cymbidium Species Based on Leaf Morpho-Anatomic Features

1. Leaf shape oblanceolate to oblong	*C. lancifolium*
1. Leaf lorate	(2)
2. Leaf margin toothed	(3)
2. Leaf margin entire	(6)
3. Leaf with petiole	*C. qiubeiense*
3. Leaf without petiole	(4)
4. Leaf base with articulate	*C. kanran*
4. Leaf base without articulate	(5)
5. Stomata sunken	*C. faberi*
5. Stomata flat	*C. serratum*
6. Leaf apex with slightly two-lobed tip	(7)
6. Leaf apex without two-lobed tip	(8)
7. Stomata amphistomatic; stomatal index largest	*C. eburneum*
7. Stomata hypostomatous	*C. mastersii*
8. The leaf midrib with shallow V-shape	(9)
8. The leaf midrib with V-shape	(10)
9. Polygonal ad-abaxial epidermal cells	*C. hookerianum*
9. Polygonal ad-abaxial epidermal cells	*C. defoliatum*
10. Flat leaf mid-rib adaxial side cross-section outline; the cuticle thickest	*C. tracyanum*
10. Semicircular leaf mid-rib adaxial side cross-section outline	(11)
11. Lacked fiber bundles; without stegmata	*C. floribundum*
11. With fiber bundles and stegmata	*C. wenshanense*
